# P-674. The Composite Hierarchical Outcomes In Respiratory infections (CHOIR) Study: An International Conjoint Analysis Survey Study of Clinician Priorities in Pneumonia

**DOI:** 10.1093/ofid/ofaf695.887

**Published:** 2026-01-11

**Authors:** Robert Dickson, Krishna Rao, Keith S Kaye, Owen Albin, Richard G Wunderink, Thomas Valley

**Affiliations:** University of Michigan, Ann Arbor, MI; Department of Internal Medicine, Infectious Diseases Division University of Michigan, Ann Arbor, Michigan, Ann Arbor, MI; Rutgers Robert Wood Johnson Medical School, New Brunswick, NJ; University of Michigan Medical School, Ann Arbor, MI; Northwestern University Feinberg School of Medicine, Chicago, IL; University of Michigan, Ann Arbor, MI

## Abstract

**Background:**

The development and use of hierarchical composite endpoints (HCEs) in pneumonia clinical trials are recommended by federal regulatory agencies, expert professional societies and pharmaceutical stakeholders alike. However, the selection and hierarchy of nonfatal outcomes in HCEs often reflect investigator opinion rather than the bedside priorities of frontline clinicians. To date, no study has systematically evaluated how clinicians prioritize nonfatal outcomes in clinical decision-making for patients with pneumonia.

**Table 1.**
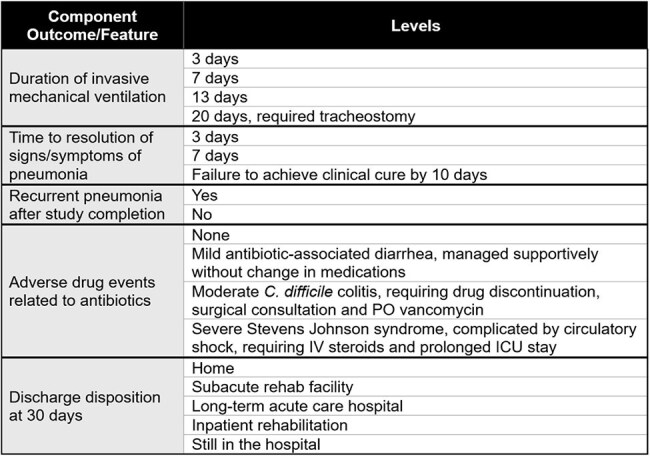
Patient Outcome Features and Levels

**Table 2.**
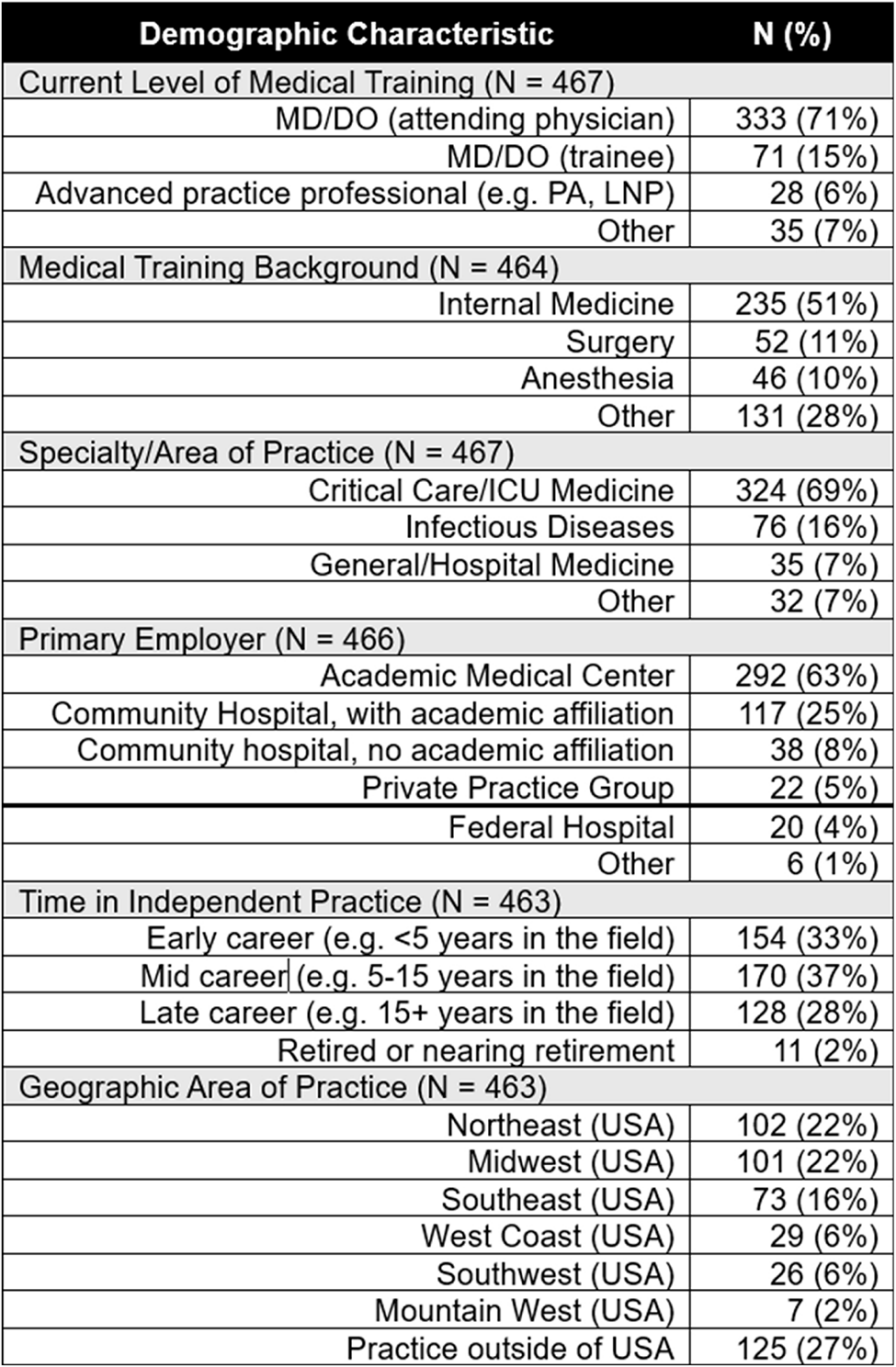
Demographic Characteristics of Survey Respondents Individual question response sizes vary per question given respondent attribution. Not all responses shown.

**Methods:**

We conducted an international web-based survey using choice-based conjoint analysis. Respondents were presented with and asked to choose a more desirable overall outcome between two hypothetical patient profiles from a ventilator-associated pneumonia (VAP) trial, each comprised of a bundle of nonfatal outcomes of varying severity (Table 1). Feature order and level selection were randomized per respondent. Bayesian hierarchical modeling was used to calculate normalized part-worth utilities and determine the relative feature importance of each nonfatal outcome. The survey targeted practicing clinicians who care for patients with VAP and was deployed via professional society listservs.

**Figure 1.**
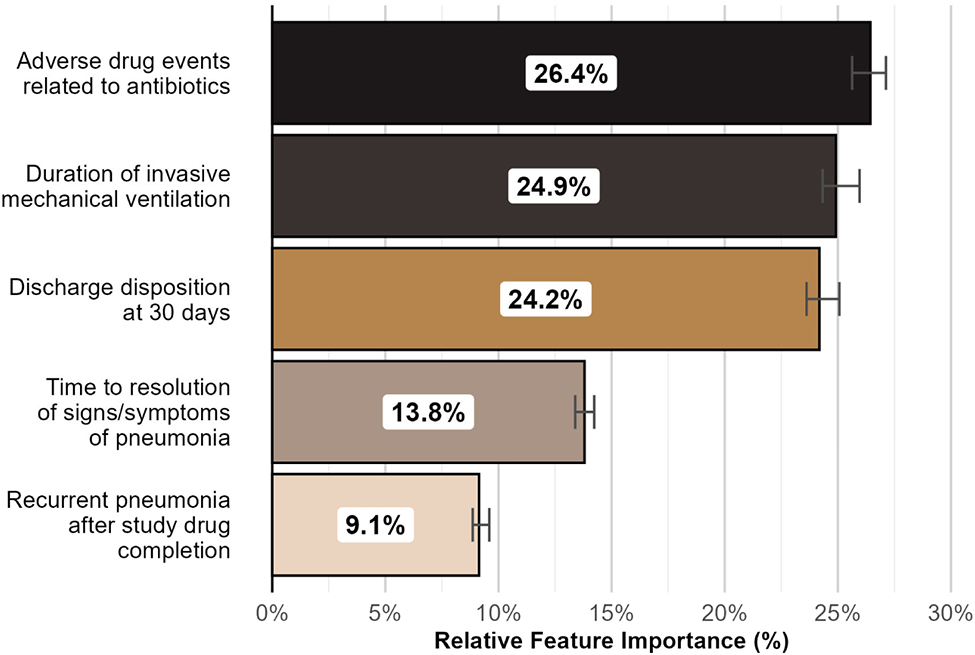
Bar Plot of Relative Feature Importance of Nonfatal Outcomes Feature importance values reflect the measure of influence of each nonfatal outcome on the desirability of the overall patient outcome and were were derived by dividing the range of part-worth utilities across a given feature's levels by the sum of all attribute part-worth utility score ranges. 95% confidence intervals derived via bootstrapping and display in error bars.

**Figure 2.**
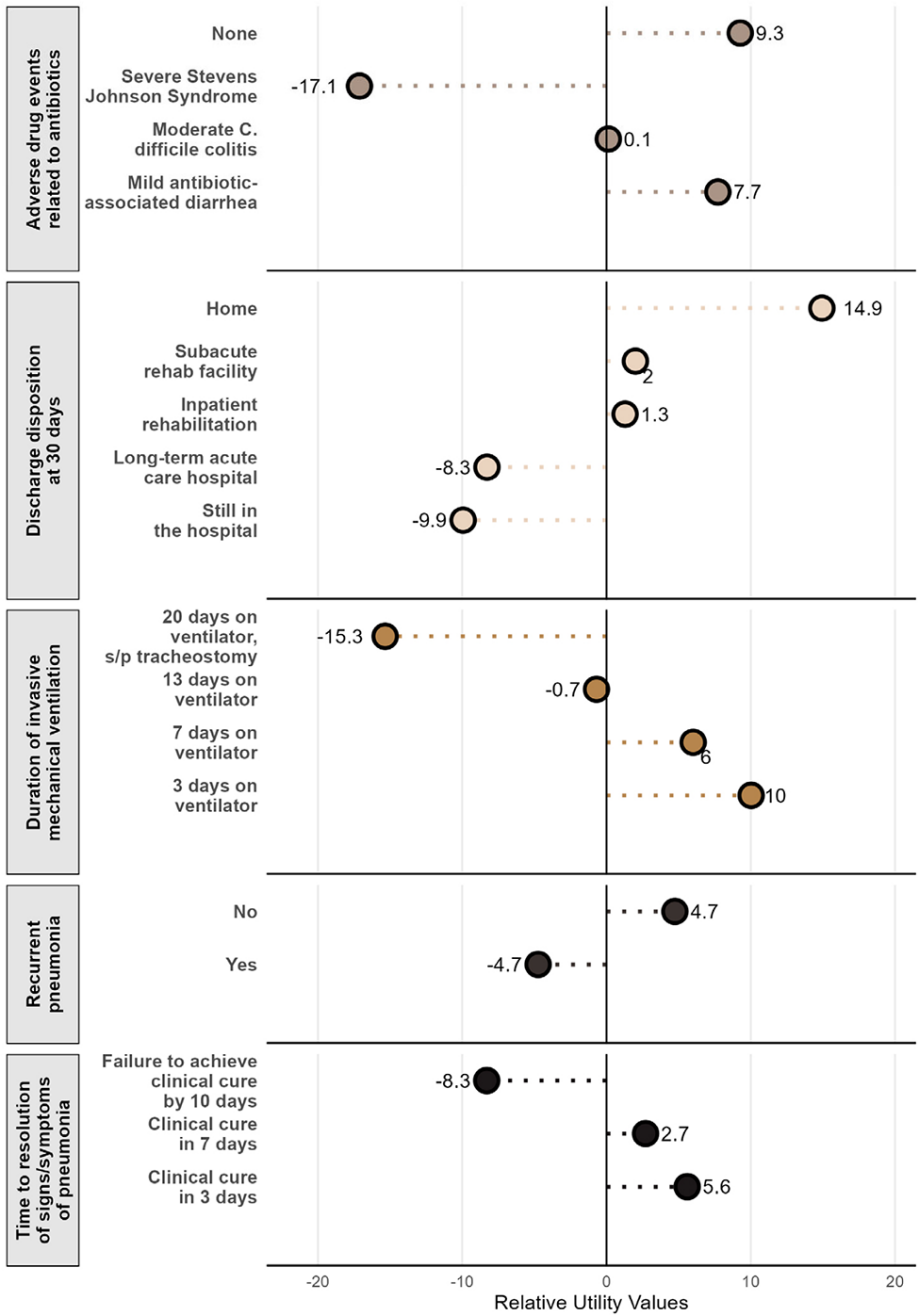
Lollipop Plot of Part-Worth Utility Scores of Nonfatal Outcomes. Relative utility values (normalized part-worth utility scores) represent the weighted value respondents assigned to each nonfatal outcome level. A higher relative utility value implies a greater preference for a given feature level and a greater weight the presence of said feature level has on respondent choice, whereas a lower/negative relative utility value implies a lower preference for a given feature level and a lower weight the presence of said feature level has on respondent choice.

**Results:**

The survey had 506 respondents and a 93% completion rate; demographics are shown in Table 2. Clinicians prioritized antibiotic-related adverse events (median feature importance 26.4% [95% CI 25.7-26.8%]), duration of invasive mechanical ventilation (24.9% [95% CI 24.4-25.9%]), and 30-day discharge disposition (24.2% [95% CI 23.6-25.1%]), over time to symptoms resolution (13.8% [95% CI 13.4-14.2%]), and pneumonia recurrence (9.2% [95% CI 8.6-9.6%]) (Figure 1). Part-worth utility scores for individual feature levels are shown in Figure 2.

**Conclusion:**

In this international conjoint analysis survey, clinicians prioritized outcomes often excluded from current pneumonia HCEs, such as discharge disposition and antibiotic-related adverse events. In contrast, they placed less value on pneumonia recurrence and time to clinical cure, despite their frequent use in existing pneumonia regulatory/comparative effectiveness trials. These findings suggest that pneumonia trial HCEs may be misaligned with the priorities of frontline clinicians.

**Disclosures:**

Krishna Rao, MD, MS, Merck & Co, Inc.: Grant/Research Support|Rebiotix Inc.: Advisor/Consultant|Seres Therapeutics: Advisor/Consultant|SUmmit Therapeutics Inc: Advisor/Consultant Keith S. Kaye, MD, MPH, AbbVie: Advisor/Consultant|GSK: Advisor/Consultant|Merck: Advisor/Consultant|Shionogi: Advisor/Consultant Owen Albin, MD, Biomerieux: Advisor/Consultant|Biomerieux: Grant/Research Support|Charles River Laboratories: Advisor/Consultant

